# Identification of independent risk factors for hypoalbuminemia in patients with CKD stages 3 and 4: the construction of a nomogram

**DOI:** 10.3389/fnut.2024.1453240

**Published:** 2024-10-31

**Authors:** Chong-Hui Wang, Meng-Han Jiang, Ji-Min Ma, Ming-Cong Yuan, Lei Liao, Hao-Zhang Duan, Dan Wang, Lian Duan

**Affiliations:** ^1^College of Pharmacy, Kunming Medical University, Kunming, China; ^2^Department of Pharmacy, Anning First People’s Hospital Affiliated to Kunming University of Science and Technology, Kunming, China; ^3^College of Pharmacy, Dali University, Dali, China

**Keywords:** CKD, hypoalbuminemia, nomogram, area under the receiver operator characteristic curve, calibration curves, decision curve analysis

## Abstract

**Background:**

Hypoalbuminemia is a common complication in patients with chronic kidney disease (CKD) and is associated with various adverse clinical events. Currently, there are few studies focused on identifying risk factors and constructing models for hypoalbuminemia in patients with CKD stages 3 and 4. This study aims to identify independent risk factors for hypoalbuminemia in patients with CKD stages 3 and 4 and construct a nomogram for predicting the risk of hypoalbuminemia in these patients.

**Methods:**

A total of 237 patients with CKD stages 3 and 4 treated at Anning First People’s Hospital from January to December 2023 were included. Univariate and bidirectional stepwise multivariate logistic regression analyses were used to identify independent risk factors for hypoalbuminemia in these patients. Based on the results of logistic regression analyses, a nomogram was constructed. The model performance was assessed using area under the receiver operator characteristic curve, calibration curves, and decision curve analysis.

**Results:**

Hemoglobin, red blood cells, serum sodium, and serum calcium were identified as independent risk factors for hypoalbuminemia in these patients. The contributions of each independent risk factor to hypoalbuminemia were visualized in a nomogram. The area under the receiver operator characteristic curve of the model was 0.819, indicating good discrimination. The calibration curves showed good agreement between predicted and observed outcomes. The decision curve analysis also verified that the model had the good clinical utility.

**Conclusion:**

Hemoglobin, red blood cells, serum sodium, and serum calcium were identified as independent risk factors of hypoalbuminemia in patients with CKD stages 3 and 4. The nomogram exhibits good discrimination, calibration, and clinical utility, offering a reliable tool for the early prediction and identification of hypoalbuminemia in these patients.

## 1 Introduction

Hypoalbuminemia is a common complication among patients with chronic kidney disease (CKD), characterized by plasma total protein levels below 60 g/L or albumin levels below 35 g/L. The mechanisms underlying hypoalbuminemia in patients with CKD are not fully understood, and multiple factors such as inadequate protein intake, protein synthesis disorders, excessive protein loss, inflammatory responses, and protein loss during dialysis contribute to its occurrence (1–3). Hypoalbuminemia is not only closely associated with malnutrition, chronic inflammation, cardiovascular diseases, and infections in patients with CKD (1, 4–6), but is also considered a predictor for the initiation of hemodialysis in patients with CKD (7). Additionally, hypoalbuminemia is recognized as a strong predictor of all-cause mortality in these patients (8). Furthermore, hypoalbuminemia and its accompanying proinflammatory state play significant roles in various diseases, not merely confined to CKD. In recent years, with the deepening of medical research, an increasing amount of evidence has emerged indicating that hypoalbuminemia and its associated proinflammatory state extensively participate in and influence the occurrence, development, and outcome of multiple diseases (9, 10). For instance, in oncology, factors such as the levels of inflammatory markers (including albumin and fibrinogen) have been confirmed as independent predictors of poor prognosis in cancer patients (9). Therefore, prediction of hypoalbuminemia in patients with CKD stages 3 and 4 can help clinicians identify and intervene in the early occurrence of hypoalbuminemia, reduce the incidence of related complications, improve the quality of life, lower the readmission rate and mortality, and delay the progression to end-stage renal disease.

In the process of establishing prediction models, most current studies typically discuss hypoalbuminemia only as a disease marker or a risk factor, such as a marker of intrinsic liver function deterioration or a strong predictor of early mortality after infective endocarditis surgery (11, 12). However, few studies have investigated hypoalbuminemia as an independent predictive outcome. Additionally, due to the complexity of clinically predicting hypoalbuminemia, there are no effective predictive tools for accurately, intuitively, and conveniently predicting hypoalbuminemia in patients with CKD stages 3 and 4.

Based on the above, we identified risk factors that may influence the occurrence of from commonly accessible hospital data. Through binary logistic regression analysis, we screened for independent risk factors that contribute to hypoalbuminemia in patients with CKD stages 3 and 4. Subsequently, we visualized the results of the logistic regression analysis to construct a nomogram for predicting hypoalbuminemia risk in these patients, providing a reliable, convenient and universally applicable tool for clinical prediction, identification and intervention.

Nomogram is a predictive tool that visualize the results of binary logistic regression models, graphically representing the probability of clinical events. Recently, nomograms have been widely used for predicting the occurrence, development, prognosis, and survival of diseases (13).

## 2 Materials and methods

### 2.1 Study design and participants

A retrospective analysis was conducted on clinical data of patients with CKD stages 3 and 4 admitted to Anning First People’s Hospital from January 2023 to December 2023. The inclusion criteria were: age > 18 years, diagnosis of CKD stages 3 and 4 according to the Kidney Disease: Improving Global Outcomes 2024 Clinical Practice Guidelines for the Evaluation and Management of Chronic Kidney Disease (14) and complete clinical data. The exclusion criteria were: incomplete clinical data, pregnancy, severe infections, severe acid–base imbalance or electrolyte disturbances and concomitant malignant tumors or other severe diseases. Based on these criteria, a total of 237 patients were included in the study. This study is a retrospective analysis focusing on patients with CKD stages 3 and 4. All information was collected from the electronic medical record system of Anning First People’s Hospital. Any data obtained about the study participants will be de-identified and kept strictly confidential, and it will be used solely for the purposes of this study. Public reports regarding the results of this research will not disclose any personal identifying information. This study has been approved by the Medical Ethics Committee of Anning First People’s Hospital (approval number: 2024-040). Due to the non-invasive and anonymous nature of retrospective studies, the authors have signed a waiver of informed consent.

### 2.2 Demographic and laboratory measurements

Demographic and clinical data collected included: age, sex, body mass index, chronic kidney disease staging (stages 3 and 4), hypertension, diabetes, cardiovascular disease, cerebrovascular disease, smoking, and drinking status. Laboratory biochemical indices included: hemoglobin (g/L), red blood cells (10^12/L), aspartate aminotransferase (U/L), alanine aminotransferase (U/L), alkaline phosphatase (U/L), total cholesterol (mmol/L), high-density lipoprotein cholesterol (mmol/L), low-density lipoprotein cholesterol (mmol/L), triglycerides (mmol/L), serum creatinine (μmol/L), blood urea nitrogen (mmol/L), serum uric acid (μmol/L), N-terminal pro-B-type natriuretic peptide (pg/ml), serum sodium (mmol/L), serum potassium (mmol/L), serum chloride (mmol/L), serum calcium (mmol/L), serum phosphorus (mmol/L), proteinuria, and carbon dioxide binding capacity (mmol/L).

### 2.3 Statistical analysis

All data were statistically analyzed using R software version 4.22. The normality of continuous variables was tested using the Shapiro–Wilk test. Continuous variables that followed a normal distribution were expressed as mean ± standard deviation and compared between groups using the *t*-test. Non-normally distributed continuous variables were expressed as median and interquartile range (IQR) and compared using the Mann–Whitney U test. Categorical variables were presented as counts (percentages) and compared using the chi-square test. Univariate logistic regression analysis was conducted for all variables, and those with *p* < 0.05 were included in a bidirectional stepwise multivariate logistic regression to identify independent risk factors for hypoalbuminemia in patients with CKD stages 3 and 4. The bidirectional stepwise multivariate logistic regression combines the advantages of both forward stepwise regression and backward stepwise regression, enabling automatic selection of variables that have significant impacts on the results during the modeling process while eliminating unimportant variables. This helps reduce the complexity of the model and improve its interpretability and generalization ability. The bidirectional stepwise multivariate logistic regression ensures that only variables with significant impacts on the results are included in the final model, thereby enhancing the interpretability of the model. The strength of prediction was quantified using odds ratios (OR) and confidence intervals (CI). Based on the multivariate logistic regression results, a nomogram was constructed using the rms package in R software. The discrimination of the model was assessed using the area under the receiver operator characteristic curve (AUC) calculated by the pROC package and visualized via receiver operator characteristic curve (ROC). Internal validation was performed with 1,000 bootstrap resamples to construct calibration curves, evaluating the calibration of the model. The clinical net benefit of the model was assessed using the rmda package to plot decision curve analysis (DCA), thus evaluating its clinical utility.

## 3 Results

### 3.1 Baseline characteristics

**Figure 1:**
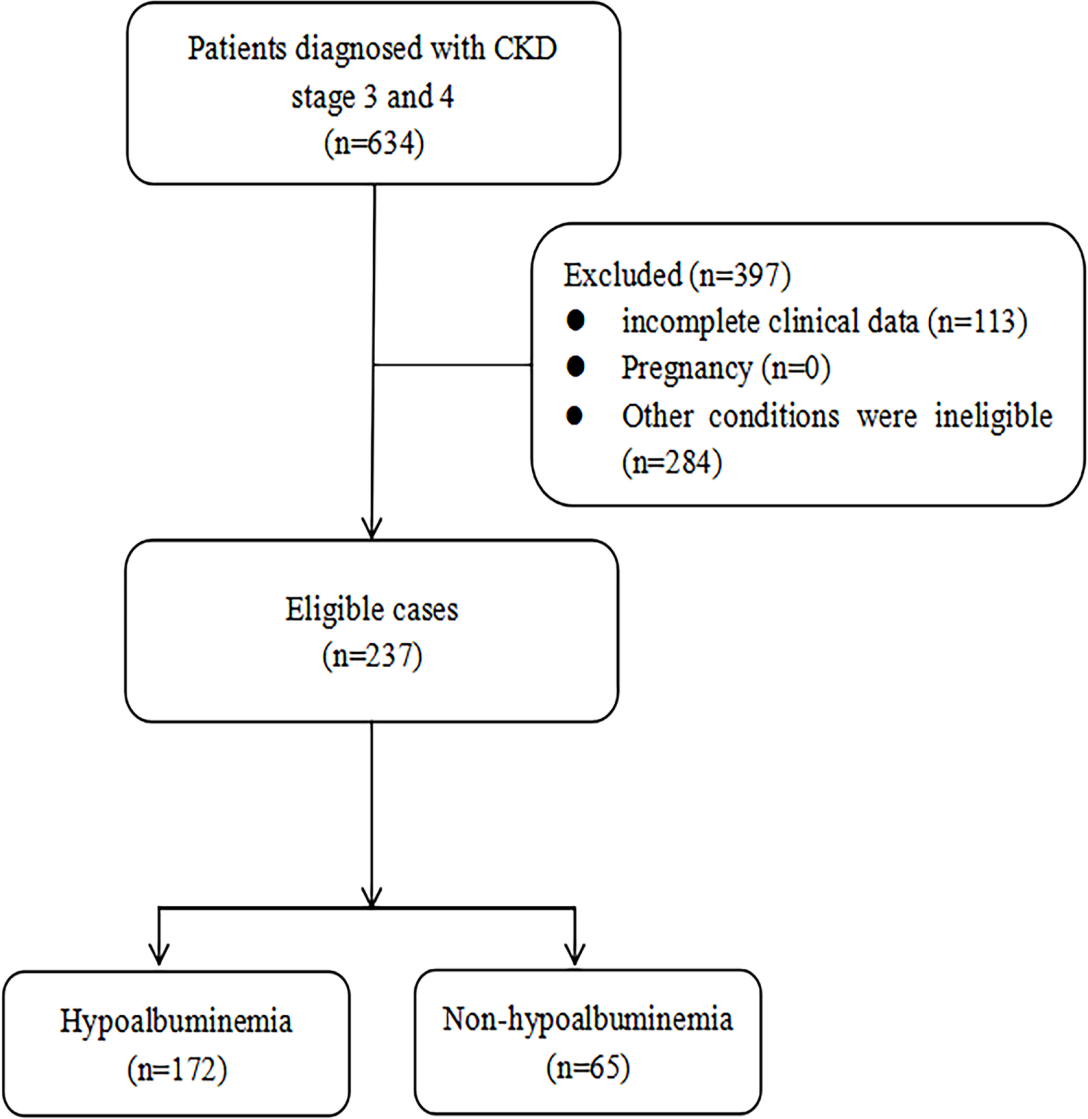
Flowchart of study inclusion.

Based on the inclusion and exclusion criteria, a total of 237 patients were included in our study (Figure 1), comprising 153 males and 84 females with a median age of 72 years (IQR: 60–80). According to the diagnostic criteria for hypoalbuminemia, the patients were divided into the hypoalbuminemia group (*n* = 65, 42 males and 23 females, median age 72 years [IQR: 66–79]) and the non-hypoalbuminemia group (*n* = 172, 111 males and 61 females, median age 71.5 years [IQR: 57.75–80]). Detailed clinical and laboratory data for both groups are presented in Table 1. Significant differences (*p* < 0.05) were observed between the two groups in body mass index, chronic kidney disease staging, proteinuria, red blood cell, hemoglobin, serum creatinine, N-terminal pro-B-type natriuretic peptide, blood urea nitrogen, serum sodium, and serum calcium.

**Table 1:** General information and clinical characteristics of participants.

Variables	Total (*n* = 237)	Hypoalbuminemia (*n* = 172)	Non-hypoalbuminemia (*n* = 65)	Statistic	*p*
Age, M (IQR),years	72.00 (60.00, 80.00)	71.50 (57.75, 80.00)	72.00 (66.00, 79.00)	*Z* = −1.16	0.246
Sex, *n* (%)				*χ*^2^ = 0.00	0.991
0	153 (64.56)	111 (64.53)	42 (64.62)		
1	84 (35.44)	61 (35.47)	23 (35.38)		
BMI, M (IQR)	23.66 (21.33, 26.30)	24.05 (21.77, 26.76)	23.24 (20.94, 24.97)	*Z* = −2.40	0.016
Chronic kidney disease staging, *n* (%)				*χ*^2^ = 6.23	0.013
3	126 (53.16)	100 (58.14)	26 (40.00)		
4	111 (46.84)	72 (41.86)	39 (60.00)		
Hypertension, *n* (%)				*χ*^2^ = 0.48	0.490
0	47 (19.83)	36 (20.93)	11 (16.92)		
1	190 (80.17)	136 (79.07)	54 (83.08)		
Diabetes, *n* (%)				*χ*^2^ = 1.37	0.241
0	142 (59.92)	107 (62.21)	35 (53.85)		
1	95 (40.08)	65 (37.79)	30 (46.15)		
Cardiovascular disease, *n* (%)				χ^2^ = 1.86	0.173
0	140 (59.07)	97 (56.40)	43 (66.15)		
1	97 (40.93)	75 (43.60)	22 (33.85)		
Cerebrovascular disease, *n* (%)				*χ*^2^ = 0.29	0.591
0	184 (77.64)	132 (76.74)	52 (80.00)		
1	53 (22.36)	40 (23.26)	13 (20.00)		
Smoking, *n* (%)				*χ*^2^ = 2.28	0.131
0	189 (79.75)	133 (77.33)	56 (86.15)		
1	48 (20.25)	39 (22.67)	9 (13.85)		
Drinking, *n* (%)				*χ*^2^ = 0.07	0.787
0	217 (91.56)	158 (91.86)	59 (90.77)		
1	20 (8.44)	14 (8.14)	6 (9.23)		
HGB (g/L), Mean ± SD	125.53 ± 25.68	130.11 ± 24.48	113.42 ± 25.02	*t* = 4.66	<0.001
RBC (10^12/L), Mean ± SD	4.15 ± 0.84	4.27 ± 0.81	3.83 ± 0.85	*t* = 3.72	<0.001
AST (U/L), M (IQR)	21.00 (17.00, 28.00)	21.00 (17.00, 27.00)	20.00 (16.00, 29.00)	*Z* = −0.52	0.601
ALT (U/L), M (IQR)	18.00 (13.00, 27.00)	18.00 (13.00, 28.00)	17.00 (11.00, 25.00)	*Z* = −0.59	0.557
ALP (U/L), M (IQR)	81.00 (65.00, 107.00)	80.50 (64.00, 103.75)	83.00 (66.00, 112.00)	*Z* = −0.48	0.628
Scr (μmol/L), M (IQR)	160.10 (131.10, 218.50)	154.95 (124.45, 203.03)	176.40 (148.60, 237.10)	*Z* = −2.88	0.004
BUN (μmol/L), M (IQR)	10.68 (7.95, 13.94)	9.98 (7.79, 13.18)	12.37 (9.20, 14.40)	*Z* = −2.55	0.011
UA (μmol/L), M (IQR)	496.00 (397.00, 626.00)	511.00 (406.00, 633.25)	480.00 (393.00, 576.00)	Z = −1.16	0.248
TC (mmol/L), M (IQR)	4.55 (3.75, 5.61)	4.49 (3.77, 5.42)	4.69 (3.60, 5.91)	*Z* = −0.81	0.416
TG (mmol/L), M (IQR)	1.57 (1.13, 2.35)	1.63 (1.19, 2.67)	1.44 (0.97, 2.16)	*Z* = −1.28	0.202
HDL-c (mmol/L), M (IQR)	1.01 (0.82, 1.23)	1.01 (0.84, 1.20)	0.96 (0.79, 1.27)	*Z* = −0.46	0.646
LDL-c (mmol/L), M (IQR)	2.50 (1.86, 3.36)	2.50 (1.88, 3.31)	2.54 (1.81, 3.36)	*Z* = −0.04	0.967
Serum sodium (mmol/L), M (IQR)	141.70 (138.90, 144.00)	142.10 (139.30, 144.20)	140.70 (137.60, 142.80)	Z = −2.87	0.004
Serum potassium (mmol/L), M (IQR)	4.31 (3.99, 4.75)	4.30 (4.02, 4.67)	4.31 (3.79, 4.89)	*Z* = −0.11	0.912
Serum chloride (mmol/L), M (IQR)	106.10 (103.10, 109.00)	105.85 (102.70, 108.73)	106.40 (104.60, 109.70)	*Z* = −1.79	0.074
Serum calcium(mmol/L), M (IQR)	2.21 (2.12, 2.30)	2.25 (2.18, 2.32)	2.11 (2.01, 2.18)	*Z* = −7.17	<0.001
Serum phosphorus (mmol/L), M (IQR)	1.20 (1.02, 1.36)	1.20 (1.03, 1.34)	1.19 (1.00, 1.37)	*Z* = −0.35	0.728
Carbon dioxide binding capacity (mmol/L), M (IQR)	22.10 (20.10, 23.70)	22.10 (20.35, 23.90)	22.10 (19.60, 23.30)	*Z* = −1.01	0.315
NT-proBNP (pg/ml), M (IQR)	690.00 (220.00, 2397.00)	393.00 (196.75, 1747.00)	1194.00 (604.00, 5315.00)	*Z* = −3.98	<0.001
Proteinuria, *n* (%)				*χ*^2^ = 4.62	0.032
0	107 (45.15)	85 (49.42)	22 (33.85)		
1	130 (54.85)	87 (50.58)	43 (66.15)		

0, no; 1, yes; 3, chronic kidney disease stage 3; 4,chronic kidney disease stage 4; T, t-test; Z, Mann–Whitney test; *χ*^2^, Chi-square test,SD: standard deviation; *p*, *p* value; M, Median; IQR, interquartile range.

AST, aspartate aminotransferase; ALT, alanine aminotransferase; ALP, alkaline phosphatase; BUN, blood urea nitrogen; HGB, hemoglobin; HDL-c, high-density lipoprotein cholesterol; LDL-c, low-density lipoprotein cholesterol; NT-proBNP, N-terminal pro-B-type natriuretic peptide; RBC, red blood cells; Scr, serum creatinine; TC, total cholesterol; TG, triglycerides; UA, serum uric acid.

### 3.2 Logistic regression analyses

**Figure 2:**
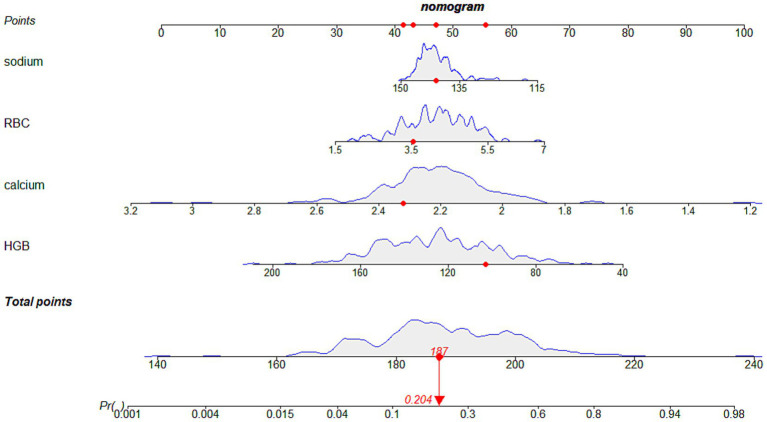
The nomogram for hypoalbuminemia prediction in patients with CKD stages 3 and 4. We could use this nomogram to predict the risk of hypoalbuminemia in patients with chronic kidney disease stages 3 and 4. For example, as shown in this image, if a patient with chronic kidney disease stage 3 or stage 4 has hemoglobin of 3.55 g/L, red blood cells of 10^12/L, serum sodium of 141 mmol/L, and serum calcium of 2.32 mmol/L, then the scores for each individual risk factor would be approximately 42, 56, 47, and 42, respectively. The total score would be around 187, corresponding to an approximate 20.4% probability of developing hypoalbuminemia. HGB, hemoglobin; RBC, red blood cells; sodium, serum sodium; calcium, serum calcium.

Univariate and bidirectional stepwise multivariate logistic regression analyses identified red blood cell (OR = 2.00, 95%CI: 0.78–5.12, *p* = 0.1473), hemoglobin (OR = 0.96, 95% CI: 0.93–0.99, *p* = 0.0169), serum sodium (OR = 0.93, 95% CI: 0.87–0.99, *p* = 0.0498), and serum calcium (OR = 0.0035, 95% CI: 0.0003–0.0433, *p* < 0.0001) as independent risk factors for hypoalbuminemia inpatients with CKD stages 3 and 4 (Table 2). Based on these independent risk factors, a nomogram for predicting the risk of hypoalbuminemia inpatients with CKD stages 3 and 4 was constructed (Figure 2). The contribution of each independent risk factor to the development of hypoalbuminemia was scored, and the total prediction score was obtained by summing these scores. The total prediction score corresponded to the predicted probability of hypoalbuminemia in patients with CKD stages 3 and 4.

**Table 2:** Univariate and multivariate logistic regression analysis in participants.

Variables	Univariate analysis		Multivariate analysis	
	*p*	OR (95%CI)	*p*	OR (95%CI)
Age, years	0.164	1.02 (0.99 ~ 1.04)		
Sex				
0		1.00 (Reference)		
1	0.991	1.00 (0.55 ~ 1.81)		
BMI	0.017	0.90 (0.83 ~ 0.98)		
Chronic kidney disease staging				
3		1.00 (Reference)		
4	0.013	2.08 (1.16 ~ 3.73)		
Hypertension				
0		1.00 (Reference)		
1	0.491	1.30 (0.62 ~ 2.74)		
Diabetes				
0		1.00 (Reference)		
1	0.242	1.41 (0.79 ~ 2.51)		
Cardiovascular disease				
0		1.00 (Reference)		
1	0.174	0.66 (0.36 ~ 1.20)		
Cerebrovascular disease				
0		1.00 (Reference)		
1	0.592	0.83 (0.41 ~ 1.67)		
Smoking				
0		1.00 (Reference)		
1	0.135	0.55 (0.25 ~ 1.21)		
Drinking				
0		1.00 (Reference)		
1	0.788	1.15 (0.42 ~ 3.13)		
HGB (g/L)	<0.001	0.97 (0.96 ~ 0.99)	0.017	0.96 (0.93 ~ 0.99)
RBC (10^12/L)	<0.001	0.52 (0.36 ~ 0.75)	0.147	2.00 (0.78 ~ 5.12)
AST (U/L)	0.449	1.01 (0.99 ~ 1.02)		
ALT (U/L)	0.477	1.00 (0.99 ~ 1.02)		
ALP (U/L)	0.093	1.00 (1.00 ~ 1.01)		
Scr (μmol/L)	0.073	1.00 (1.00 ~ 1.01)		
BUN (μmol/L)	0.032	1.05 (1.01 ~ 1.10)		
UA (μmol/L)	0.215	1.00 (1.00 ~ 1.00)		
TC (mmol/L)	0.118	1.14 (0.97 ~ 1.34)		
TG (mmol/L)	0.829	1.02 (0.87 ~ 1.20)		
HDL-c (mmol/L)	0.700	1.16 (0.54 ~ 2.47)		
LDL-c (mmol/L)	0.993	1.00 (0.79 ~ 1.27)		
Serum sodium (mmol/L)	0.025	0.93 (0.87 ~ 0.99)	0.050	0.93 (0.87 ~ 0.99)
Serum potassium (mmol/L)	0.376	1.17 (0.82 ~ 1.67)		
Serum chloride (mmol/L)	0.082	1.06 (0.99 ~ 1.12)		
Serum calcium (mmol/L)	<0.001	0.00 (0.00 ~ 0.02)	<0.001	0.00 (0.00 ~ 0.04)
Serum phosphorus (mmol/L)	0.815	0.91 (0.42 ~ 1.99)		
Carbon dioxide binding capacity (mmol/L)	0.099	0.93 (0.85 ~ 1.01)		
NT-proBNP (pg/ml)	0.101	1.00 (1.00 ~ 1.00)		
Proteinuria				
0		1.00 (Reference)		
1	0.033	1.91 (1.05 ~ 3.46)		

0, no; 1, yes; 3, chronic kidney disease stage 3; 4, chronic kidney disease stage 4; *p*, *p* value; OR, Odds Ratio; CI, Confidence Interval.

AST, aspartate aminotransferase; ALT, alanine aminotransferase; ALP, alkaline phosphatase; BUN, blood urea nitrogen; HGB, hemoglobin; HDL-c, high-density lipoprotein cholesterol; LDL-c, low-density lipoprotein cholesterol; NT-proBNP, N-terminal pro-B-type natriuretic peptide; RBC, red blood cells; Scr, serum creatinine; TC, total cholesterol; TG, triglycerides; UA, serum uric acid.

### 3.3 Model performance and validation

**Figure 3:**
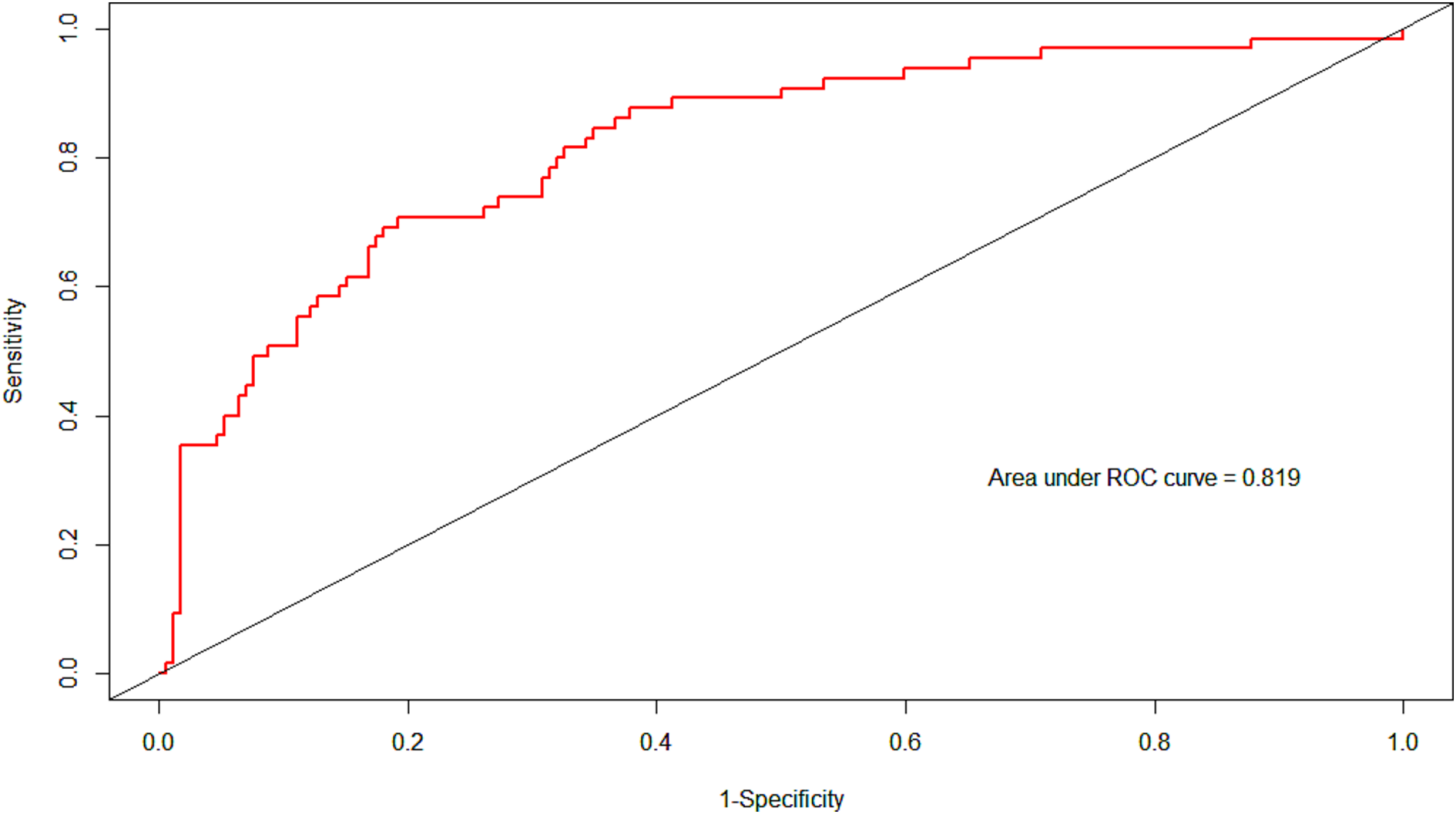
The ROC of the models. Area under the ROC was 0.819. ROC, receiver operator characteristic curve.

**Figure 4:**
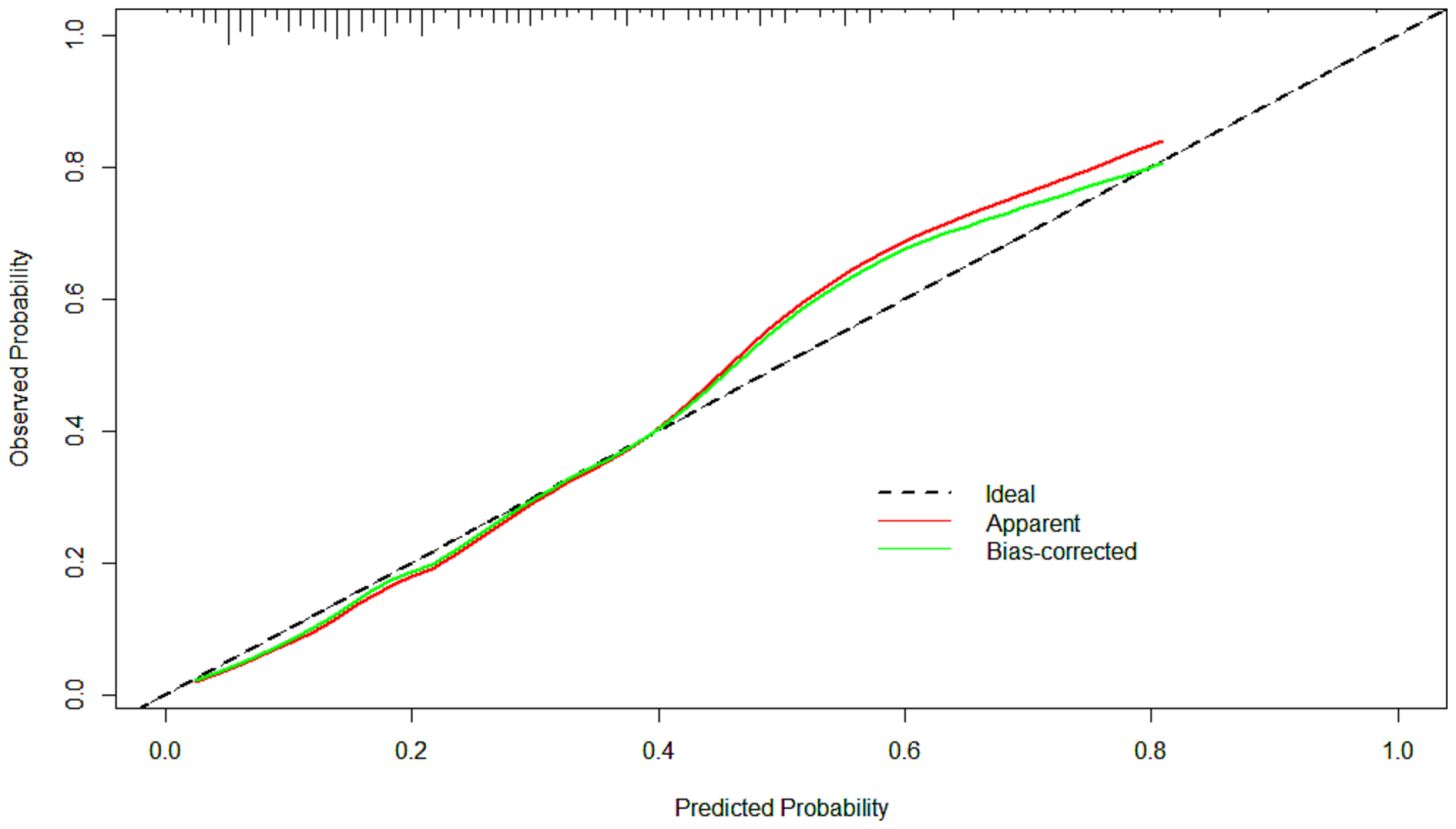
The calibration curves of the model. The clinical utility of the model was demonstrated by the calibration curves with the bootstrap (*B* = 1,000) technique. The calibration curves showed good agreement between the predicted and observed outcomes.

**Figure 5:**
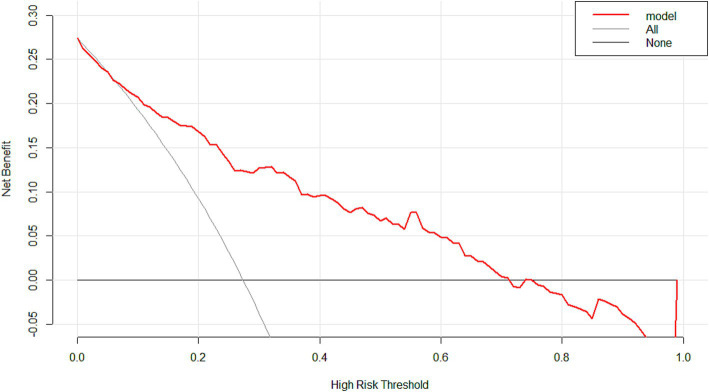
The decision curve analysis of the model. With a wide range of threshold probabilities, the model indicated high clinical utility.

The ROC curve of the model showed good discrimination with an AUC of 0.819 (95% CI: 0.757–0.880) (Figure 3). Internal validation with 1,000 bootstrap resamples and calibration curves analysis indicated good agreement between the predicted and observed outcomes, demonstrating good calibration of the model (Figure 4). The decision curve analysis showed net clinical benefits across a wide range of threshold probabilities, indicating high clinical utility of the model (Figure 5).

## 4 Discussion

According to the result of our logistic regression analyses, hemoglobin, red blood cells, serum sodium, and serum calcium were identified as independent risk factors. Our study also developed a nomogram to predict the risk of hypoalbuminemia in patients with CKD stages 3 and 4. Our validation showed that the nomogram had good performance and clinical utility.

Hemoglobin serve as crucial indicators reflecting the risk of hypoalbuminemia occurrence, with malnutrition being a common manifestation in affected patients (4) and previous studies have indicated the use of hemoglobin as a criterion for assessing malnutrition (15). Therefore, hemoglobin levels are important indicators reflecting the risk of hypoalbuminemia occurrence. Clinically, close attention to hemoglobin levels and variability in patients with CKD is necessary. Handelman found that when hemoglobin levels in patients with CKD fall below the target range (11–12.5 g/dL), there is an increase in hospitalization and mortality rates (16). Moreover, the fluctuation in hemoglobin levels in patients with CKD is directly proportional to the occurrence of adverse clinical events (17). Recommendations from The National Institute for Health and Clinical Excellence and The Trial to Reduce Cardiovascular Events with Aranesp Therapy study suggest maintaining hemoglobin levels in patients with CKD between 110 and 120 g/L to reduce cardiovascular events and mortality risk (18, 19). It is not only essential to avoid low hemoglobin levels but also to prevent hemoglobin variability. Strategies such as the use of erythropoietin, iron supplementation, blood transfusions, and enhancing nutritional intake can improve low hemoglobin levels in patients with CKD (20–22). However, maintaining hemoglobin levels within a reasonable and stable range while avoiding hemoglobin variability remains a significant challenge in clinical practice due to various complex factors affecting hemoglobin levels in patients with CKD, such as inflammation, blood loss, and inadequate protein intake.

Red blood cells are also identified as independent risk factors for hypoalbuminemia in patients with CKD stages 3 and 4. Firstly, similar to hemoglobin, malnutrition, one of the manifestations of hypoalbuminemia, can affect red blood cell levels (23). Secondly, factors such as toxin accumulation, shortened red blood cell lifespan, and insufficient erythropoietin secretion due to CKD-related reasons suggest a close relationship between red blood cell levels and hypoalbuminemia and kidney disease progression in patients with CKD (24). Additionally, Zhang-Zhe Peng’s study has shown that elevated levels of red blood cell markers such as adenosine monophosphate-activated protein kinase, 2,3-biphosphoglycerate, and 50% of hemoglobin binding to oxygen may play compensatory roles, promoting renal tissue oxygenation, reducing tissue damage, and delaying CKD progression (25). Therefore, close monitoring of red blood cell levels in patients with CKD is essential in clinical practice.

Lower serum sodium levels in patients with CKD stages 3 and 4 are associated with a higher risk of hypoalbuminemia. Studies have indicated that hyponatremia may be a marker of protein-energy wasting and low solute intake (26, 27), which is consistent with our results. Patients with CKD are prone to hyponatremia due to impaired sodium reabsorption in the kidneys and the use of diuretics (28). Hyponatremia can lead to imbalance, decreased bone density, increased risk of fractures, and toxicity to the brain, heart, and muscular systems (29–33). Additionally, low serum sodium levels may increase the risk of infections (34). Although some studies have suggested a higher risk of mortality with lower serum sodium levels and a lower risk with higher serum sodium levels (26), considering the long-term effects of serum sodium level changes over time, both lower and higher serum sodium levels (<138 and ≥ 144 mEq/L, respectively) are associated with increased mortality risk (35). Therefore, maintaining stable serum sodium levels in patients with CKD is crucial to prevent hypoalbuminemia, further deterioration of kidney function, and other adverse events. Close monitoring of serum sodium levels in patients with CKD in clinical practice, early prediction, and prevention of serum sodium abnormalities, and timely intervention and correction for patients with CKD with serum sodium abnormalities are necessary to avoid other adverse events.

Hypocalcemia often accompanies hypoalbuminemia because the total serum calcium concentration is the sum of ionized calcium and calcium bound to albumin (36). When albumin levels decrease, it affects calcium binding, resulting in decreased serum calcium levels. Therefore, when serum calcium levels decrease, hypoalbuminemia may occur. On the other hand, factors such as decreased synthesis of 1,25-hydroxyvitamin D in patients with CKD, hyperphosphatemia, and gastrointestinal lesions can lead to hypocalcemia (37–39). Studies have shown that hypocalcemia is associated with osteoporosis, cardiovascular events, and increased mortality rates in patients with CKD (40, 41). Additionally, according to a study by Chang-Seong Kim et al., serum calcium levels in patients with CKD are closely related to their estimated glomerular filtration rate levels (42). Another study, including African American and Hispanic participants in the Chronic Renal Insufficiency Cohort, showed that hypocalcemia is one of the complications of patients with estimated glomerular filtration rate < 20 mL/min/1.73m^2^ (43), indicating a close relationship between hypocalcemia and CKD progression. In clinical practice, more attention should be paid to serum calcium levels in patients with CKD stages 3 and 4 to maintain stable serum calcium levels, reduce the occurrence of adverse clinical events, and delay further deterioration of kidney function.

This study has several limitations. Firstly, the dataset used in this study is relatively small. Although model validation showed good performance, more data are still needed to train and validate the model further, our main task next will be to seek higher-quality research participants with a larger sample size, in order to reduce the potential interference of objective data factors and further explore its effectiveness in practical applications. Secondly, due to the source of the dataset, some potentially relevant risk factors were not collected. Although we have included known confounding factors in our analysis based on previous research literature, other unidentified factors may also impact our outcomes. However, we will actively work to improve and supplement additional information that may be related to hypoalbuminemia, aiming to provide more precise guidance for clinical treatment. Finally, this study is a single-center retrospective study, lacking external validation and prospective results to provide guiding information. In future research, one of our directions will be to conduct multicenter external validation based on large-sample real-world data or more comprehensive databases, to further expand the data and validate the model performance.

In conclusion, through univariate and bidirectional stepwise multivariate logistic regression analysis, we identified hemoglobin, red blood cells, serum sodium, and serum calcium as four independent risk factors for hypoalbuminemia in patients with CKD stages 3 and 4. Based on the logistic regression results, a nomogram was constructed. The model, validated with good discrimination, calibration, and clinical utility, can provide a reliable, convenient and universally applicable tool for early identification and intervention of hypoalbuminemia in patients with CKD stages 3 and 4. Therefore, for all patients in hypoalbuminemia with CKD stages 3 and 4, hemoglobin, red blood cell count, serum sodium, and serum calcium should be included as routine tests. Subsequently, the pre-established nomogram could be utilized to rapidly assess their risk of hypoalbuminemia, enabling early risk identification. For patients who have already exhibited abnormalities in any one or more of these indicators, the monitoring frequency should be increased, with regular rechecks of these parameters. The nomogram should also be employed for dynamic risk assessment, allowing for swift reactions to changes in disease status or adjustments to treatment plans. Furthermore, clinicians can tailor intervention plans based on the nomogram assessment results, in conjunction with the patient’s individual circumstances, to minimize the risk of hypoalbuminemia and enhance the overall health status of these patients.

## Data Availability

The original contributions presented in the study are included in the article/Supplementary material, further inquiries can be directed to the corresponding author.
